# Intracellular glycogen accumulation by human gut commensals as a niche adaptation trait

**DOI:** 10.1080/19490976.2023.2235067

**Published:** 2023-08-01

**Authors:** Maria Esteban-Torres, Lorena Ruiz, Valerio Rossini, Ken Nally, Douwe van Sinderen

**Affiliations:** a APC Microbiome Ireland, University College Cork, Cork, Ireland; b School of Microbiology, University College Cork, Cork, Ireland; c Department of Microbiology and Biochemistry of Dairy Products, Instituto de Productos Lácteos de Asturias, IPLA-CSIC, Villaviciosa, Spain; d Functionality and Ecology of Benefitial Microbes (MicroHealth Group), Instituto de Investigación Sanitaria del Principado de Asturias (ISPA), Oviedo, Asturias, Spain; e School of Biochemistry and Cell Biology, University College Cork, Cork, Ireland

**Keywords:** glycogen metabolism, commensal bacteria, gut microbiota, colonization factors, bacteria-host interactions, microbiome, carbohydrate-active enzymes

## Abstract

The human gut microbiota is a key contributor to host metabolism and physiology, thereby impacting in various ways on host health. This complex microbial community has developed many metabolic strategies to colonize, persist and survive in the gastrointestinal environment. In this regard, intracellular glycogen accumulation has been associated with important physiological functions in several bacterial species, including gut commensals. However, the role of glycogen storage in shaping the composition and functionality of the gut microbiota offers a novel perspective in gut microbiome research. Here, we review what is known about the enzymatic machinery and regulation of glycogen metabolism in selected enteric bacteria, while we also discuss its potential impact on colonization and adaptation to the gastrointestinal tract. Furthermore, we survey the presence of such glycogen biosynthesis pathways in gut metagenomic data to highlight the relevance of this metabolic trait in enhancing survival in the highly competitive and dynamic gut ecosystem.

## GRAPHICAL ABSTRACT

**Figure:**
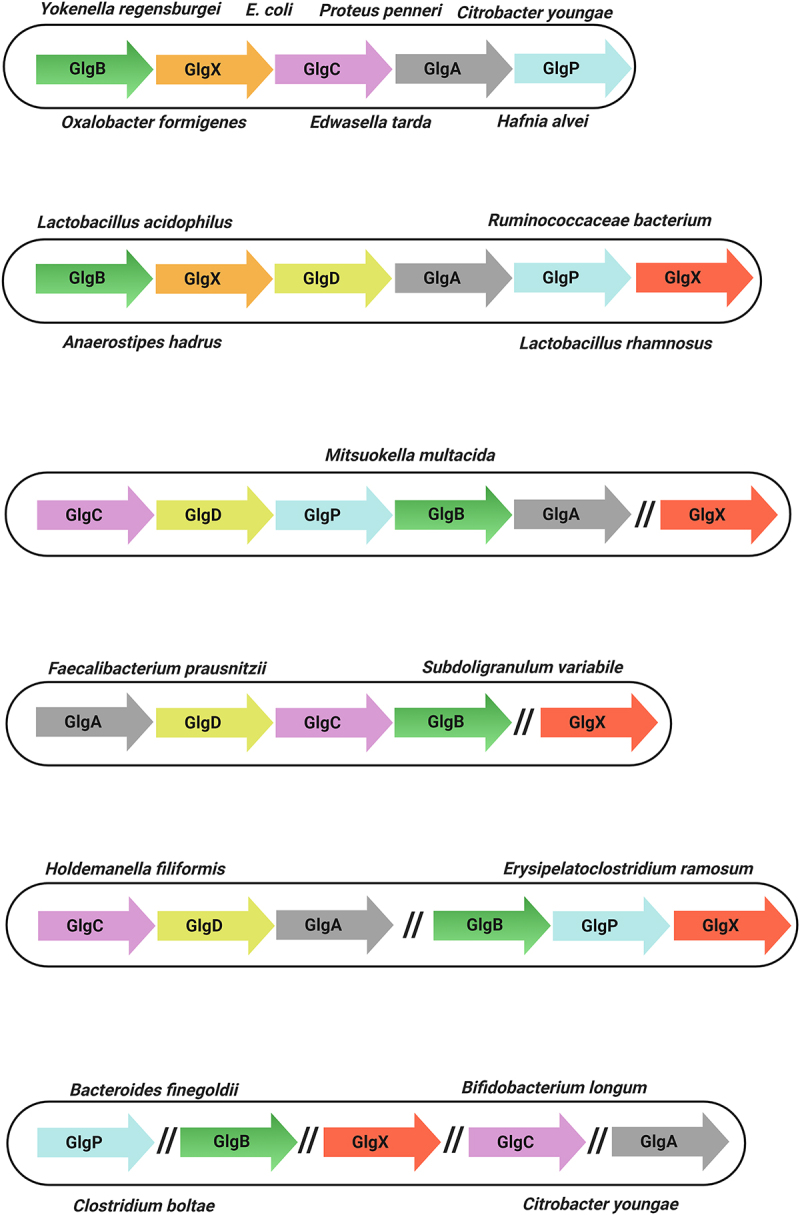

## Introduction

The human gastrointestinal tract (GIT) contains a vast, complex and dynamic microbial community, termed the gut microbiota.^1,2^ This microbial population acts as a key contributor to host metabolism and physiology, and for this reason impacts on host health.^3–5^ For example, elements of the gut microbiota stimulate proliferation and differentiation of intestinal epithelial cells (IECs) assuring efficient nutrient absorption, thereby positively contributing to human metabolism.^6^ Other beneficial metabolic functions of the gut microbiota include: (i) regulation of iron, copper, magnesium and manganese availability,^7,8^ (ii) the fermentative conversion of non-digestible food components such as dietary fibers into short chain fatty acids (SCFAs), that act as an energy source for IECs and elicit immune modulatory effects on T_regs_ and resident macrophages, (iii) the degradation of toxic compounds, and (iv) the production of vitamins (for example vitamin K and certain B vitamins).^9–12^ Furthermore, the presence of commensal bacteria has been shown to provide a barrier to intestinal infections.^13,14^

Gut microbiota studies are currently highly topical in the fields of microbiology, biotechnology, immunology and clinical medicine, while they have also raised awareness of the importance of intestinal health among the general public. Numerous primary research publications and reviews have covered aspects of the gut microbiota and its potential role in supporting and promoting human health, especially during early life,^15^ a period during which the microbiota clearly contributes to the development of the infant gut and its associated immune system.^16–20^ The gut microbiota is also known to impact on the risk of contracting certain diseases that are typically associated with adult life, such as cardiometabolic disorders, inflammatory bowel diseases, neuropsychiatric diseases and cancer.^3^ Therefore, study of the gut microbiota and its associated host benefits holds a lot of promise in terms of promoting human health and well-being.

The human colon is one of the most densely populated microbial habitats known.^21,22^ Bacteria make up the majority of living microbes present in the human GIT and by inference are believed to be the main contributors to the metabolic activities that take place in the gut.^23,24^ Bacterial gut inhabitants mainly belong to the phyla Bacteroidota, Bacillota, Pseudomonadota, Actynomicetota, Fusobacteriota and Verrucomicrobiota.^23,25,26^ It is generally accepted that microbial colonization of the GIT commences at birth; the composition of this early human gut microbiota demonstrates remarkable dynamism that gradually develops complex metabolic functions.^27^ In contrast, in adults the microbiota is more stable and resilient, though may fluctuate in response to environmental factors, while maintaining core taxa for many years.^28^

Determining the forces that shape our gut microbiota is fundamental to our understanding of the functioning of these intestinal communities.^2^ Central to this goal is gaining detailed information on the ecology of our gut microbiota and the mechanisms by which the individual components of this microbiota first establish themselves and then persist in their particular niche.^29^ The intracellular accumulation of glucose-containing polymers, such as glycogen, is an energy storage that has been associated with important physiological functions in several bacterial species. However, its role in influencing the presence and functionality of (specific components of) the gut microbiota in its habitat has not yet been thoroughly addressed.

Here, we critically review recent insights into the enzymatic machinery that certain gut bacteria use to accumulate and subsequently degrade intracellular glycogen, and discuss how this metabolic ability may facilitate colonization of the gut environment. We furthermore perform an *in silico* survey for the presence of genes that encode glycogen biosynthesis-associated enzymes in gut metagenomic libraries to highlight the prevalence of this metabolic trait as a possible fitness marker in the highly competitive and dynamic gut niche.

## Human gut microbiota and its metabolic adaptation to its natural environment

Commensal bacteria inhabiting the gut ecosystem are believed to require specific adaptations to this particular ecological niche^23^. The establishment of intricate and dynamic interactions between the human host and its inhabiting microbes is necessary for these gut commensals to survive within the GIT, obtain nutrients and reproduce, and is believed to be beneficial for both partners^30^. The GIT is rich in macromolecules that can serve as nutrients for these gut microorganisms and therefore represent a secure habitat at a constant and optimal temperature where microbes can establish and multiply^31^. Although many members of the gut microbiota cooperate, there is also intense competition for space and resources within the GIT.^30^

It is generally accepted that host diet is one of the main drivers that shape gut microbiota composition.^17,32,33^ Put in simple terms, available nutrients determine whether or not a microorganism is able to establish and persist in the gut.^29,34^ Carbohydrate fermentation is a core activity of the human gut microbiota, as carbohydrates represent a major energy and carbon source in the gut.^35^ The mammalian GIT has different compartments with diverse nutrient and environmental conditions and, consequently, different microbial communities. The small intestine houses microorganisms that utilize simple sugars, while in the colon, the bacterial inhabitants are more specialized toward complex dietary fiber fermentation^29^ and also host glycans such as mucin-associated carbohydrates.^36^ Accordingly, a plant-based, polysaccharide-rich diet favors expansion of those organisms that can metabolize such dietary fibers either directly or through cross-feeding activities^37^. Bacterial glycan fermentation generally produces beneficial metabolites in the form of SCFAs^9^. Acetate, propionate, and butyrate are the three main SCFAs, and each of these play different roles in human physiology.^9,38–40^

Metabolic adaptation describes the ability to metabolically switch from one substrate to another, a property that is essential for the fitness and survival of microorganisms in competitive environments such as the GIT.^41,42^ In the mammalian gut environment, nutrient availability is constantly fluctuating and saccharidic substrates that support bacterial growth are not continually available and if so, they are typically present in limiting amounts.^43^ The model bacterium *Escherichia coli*, as an example of a gut commensal, exhibits metabolic adaptation through its ability to use a wide range of dietary sugars in order to ensure durable gut colonization.^42–44^ Similarly, gut bacteria belonging to the genera *Bacteroides*, *Bifidobacterium* and *Ruminococcus* are capable of degrading complex carbohydrates.^45,46^ For example, in a complex nutrient environment, such as that resulting from a high fiber diet, human gut-associated *Bacteroides* species can switch their metabolism to suit available glycan substrates. However, a microorganism can still persist in the gut if it is able to utilize some of the available nutrients more efficiently than its competitors.^29^

In order to cope with life in a highly competitive nutrient-limiting or nutrient-fluctuating environment, certain bacteria, including gut commensals, have developed the ability to create intracellular energy stores, such as glycogen. This polymer is accumulated under certain conditions thanks to a dedicated enzymatic machinery to serve as a major energy reserve in bacteria that may underpin successful gut colonization and survival.^43,47,48^

### Box 1: Colonization factors in gut commensal bacteria

**Figure 1.**
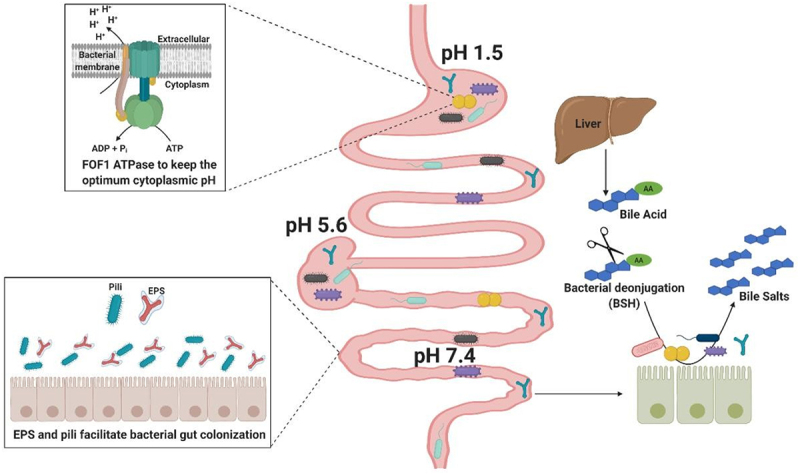
Colonization Factors in gut commensal bacteria.

To bestow beneficial activities on a human host through host-microbe mutualistic interactions, symbionts and probiotic commensals need to establish themselves, at least temporarily, in the gut environment.^49^ Thus, investigation of the mechanisms that enable these microbes to colonize, stay alive and be metabolically active in the GIT is of key importance in order to fully understand their functions.^50^ Apart from the ability of gut bacteria to metabolize dietary or host-derived compounds, other colonization factors have been described (Figure 1). These include mechanisms such as amino acid decarboxylases or the F_0_F_1_-ATPase, which allow bacteria to tolerate fluctuating and low pH (ranging from 6.5 to 7.5 in the mouth to 1.5–6 in the stomach, and from 5.7 to 7.4 in the small intestine, cecum and colon).^51,52^ In the small intestine, bacteria need to survive exposure to toxic bile, a fluid which is predominantly composed of primary and conjugated bile acids (BAs), and which acts as a potent detergent, essential for fat digestion and absorption.^53^ Many gut bacterial inhabitants use hydrolytic enzymes to protect themselves from BA toxicity.^54–58^ Production of surface exopolysaccharides (EPS), biofilms and efflux pumps have also been associated with providing bile tolerance in some bacterial species.^59–62^

Schematic representation of some of the molecular and cellular mechanisms that commensal bacteria evolved to survive in the gastrointestinal tract. (Top left panel) To tolerate the wide range of pH during the transit through different gut locations, bacteria use an ATP-driven proton pump to keep an optimum cytoplasmic pH. (Bottom left panel) Surface exopolysaccharide (EPS), and pili produced by several commensal bacteria have been shown to be essential for their adherence and colonization in the GIT. (Right panel) Bile is synthesized in the liver and then secreted into the duodenum. Bile is made of primary bile acids (BAs) and is toxic to bacteria. To protect themselves from BA-mediated toxicity several commensal bacteria use hydrolytic enzymes to deconjugate BAs.

Adhesion to intestinal epithelial cells, mucus, or components of the extracellular matrix represents an additional important bacterial colonization factor.^63–66^ Adhesion to the intestinal epithelium is considered crucial for commensal bacteria to facilitate gut establishment, thereby competing with and thus limiting colonization by intestinal pathogens.^67^ EPS appears to play an important role in bacterial interaction with the intestinal mucosa, although the precise mechanism of this interplay is still poorly understood.^68^ The presence of EPS surrounding the bacterial surface can reduce adherence to intestinal cells thereby negatively impacting on intestinal colonization.^69–72^ whereas others have demonstrated that EPS-producing bacteria are better at tolerating the gastrointestinal transit and can persist longer in the GIT.^59,73^ In addition to EPS, other extracellular structures, such as pili are implicated in adhesion processes.^74,75^ For example, sortase-mediated and Tad (tight adherence) pili have been identified in several species of commensal bacteria, including lactobacilli and bifidobacteria, and shown to be important for their adherence, gut colonization and host cell proliferation.^76–82^

## Glycogen storage by members of the gut microbiota

Energy storage in the form of branched glucose polymers is clearly an ancient ability and may have been present in the last universal common ancestor of all present life.^83^ Glycogen and other related polyglucose compounds share a common structure composed of glucose monomers that are connected by α-1,4- and α-1,6-linkages, and are among the preferred carbon sources of many gut commensals. Various bacteria in the GIT environment possess the enzymatic machinery to utilize these dietary polysaccharides as an extracellular carbon and energy source, as has been addressed in several reviews.^84,85^ Certain bacteria, employing products of the degradation of these polysaccharides, or other carbohydrates, are capable of synthesizing *de novo* glycogen-like polymers intracellularly (glycogen, starch or maltodextrin).^86^ However, relatively little attention has been paid to the physiological role of these intracellular energy storage polymers, generally referred to as glycogen, or the molecular pathways and the specific enzymes required for intracellular biosynthesis and subsequent degradation. Herein, we discuss intracellular glycogen metabolism under the terms glycogen accumulation/degradation to describe such processes, unless otherwise indicated.

Bacterially produced glycogen is a water-soluble polymer consisting of chains of α-1,4-linked glucose units that contain regular α-1,6-linked branches^87^ with an estimated average molecular weight between 10^7^ and 10^8^ Daltons.^48,88^ Glycogen branching is important for fast response to metabolic needs, because both synthesis as well as degradation of the glycogen polymer occur from the non-reducing ends of the α-1,4 chains. Thus, highly branched glycogen has a higher number of “ends” per volume, while branching also increases glycogen solubility.^83^ Due to its large molecular mass, high solubility and highly branched structure, glycogen represents an efficient form of energy and carbon storage with little effect on the internal osmolarity of cells.^86,88^

Several early studies have described accumulation of glycogen in bacteria. This characteristic was thoroughly studied in *E. coli*.^89,90^ Using spectrophotometry, Levine and colleagues discovered that under certain cultivation conditions a characteristic set of absorption bands which appeared in the infrared spectrum of this enteric bacterium, were due to a glycogen-like polysaccharide.^91^ These authors also showed marked variability in intracellular glycogen levels among different enteric strains and upon growth on different carbon sources,^91^ with *E. coli* even accumulating intracellular glycogen granules.^89^ A study carried out in *Enterobacter aerogenes* (previously *Aerobacter aerogenes*) demonstrated that utilization of degradation products of intracellular storage polymers supports bacterial survival under certain challenging conditions *in vitro*.^92^ Another classic publication provides further information on the role of glycogen as a bacterial energy and carbon reserve, though being dependent on the bacterial species and nature of the carbon and energy source in the environment.^93^ In *Bacteroides fragilis*, the intracellular polysaccharide was shown to be degraded rapidly when glucose was in short supply and fatty acids accumulated in the medium.^94^ Thus, the concept of intracellular polysaccharide accumulation in (gut-associated) bacteria was established in the early 60s.^95,96^

At that time, findings suggested that certain microorganisms, e.g. *Pseudomonas aeruginosa*, do not synthesize any specific reserve compound.^97^ More recently, it has been conclusively established that the ability to produce and accumulate intracellular α-glucan polysaccharides such as glycogen represents a feature of many, but not all bacteria.^98^ Nonetheless, intracellular glycogen accumulation has been observed in more than 50 bacterial species, including Gram-positive and Gram-negative bacteria, as well as archaebacteria.^48,87^ Indeed, glycogen storage is ubiquitous among enteric bacteria, such as *En. aerogenes*, *E. coli*, *Lactobacillus acidophilus*, *Edwardsiella tarda, Hafnia alvei*, several species of *Bacillus*, and *Streptococcus*, and various ruminal bacteria (Preiss 2006) (Table 1). Furthermore, some of these glycogen-accumulating bacteria are gut pathogens, such as *Clostridium botulinum*, several species of *Salmonella* and *Vibrio cholerae*. As we will discuss below, glycogen accumulation in both pathogenic and nonpathogenic bacteria is associated with increased gut survival and tolerance to gut-associated stress conditions.

**Table 1.** Intracellular accumulation of glycogen reported in gut-related bacteria.

	Habitat	Genome size (Mb)^a^	GC (%)^b^	Role of intracellular glycogen storage	References
*Acetivibrio cellulolyticus (synonym Hungateiclostridium cellulolyticum)*	Sewage	6.16	35.5	Survival in starvation conditions	Patel and Breuil (1981)^99^
*Aeromonas hydrophila*	Aquatic environments; animal and human (gut) pathogen	4.74	61.5	Survival in low carbohydrate environment	Shaw and Squires (1980)^100^
*Aerobacter aerogenes (Enterobacter aerogenes, Klebsiella aerogenes)*	Human gut (opportunistic pathogen)	5.28	54.8	Survival in unfavorable environments	Strange et al. (1961)^92,101^
*Bacillus megaterium*	Soil; human gut	5.34	38.1	Carbon and energy reserve	Barry et al. (1952)^102^
*Bacillus subtilis*	Soil; water; vegetables; human and animal gut	4.46	43.4	Sporulation	Kiel et al. (1964)^103^
*Bacteroides fragilis*	Mammal gut (human opportunistic pathogen);	5.21	43.2	Carbon and energy supply under low carbohydrate level conditions	Lindner et al (1979)^94^
*Clostridium botulinum*	Soil aquatic sediments; improperly preserved foods; animal and human gut (pathogen)	4.39	28	ND	Whyte and Strasdine (1972)^104^
*Corynebacterium glutamicum*	Soil; some species of *Corynebacterium* are members of the human gut microbiota	3.31	53.8	Cell shape and osmoprotection	Seibold et al., (2007), Seibold and Eikmanns (2013), Seibold et al., (2011)^47,105,106^
*Escherichia coli*	Gut of humans and other warm- blooded animals (some species are pathogenic)	4.64	50.8	Energy maintenance during metabolic adaptation and colonization	Holme et al., (1956), Fung et al., (2013), Morin et al., (2017), Jones et al., (2008), Wang et al., (2019), Wang et al., (2020)^107–109^
*Fibrobacter succinogenes*	Rumen	3.84	48		Stewart et al., (1981), Gaudet et al., (1992)^110,111^
*Lactobacillus acidophilus*	Human oral, gastrointestinal, and vaginal microbiota	2.01	34.7	Gut fitness and retention	Goh and Klaenhammer (2013), Goh and Klaenhammer (2014)^86,112^
*Megasphaera elsdenii*	Rumen	2.5	52.8	ND	Cheng et al., (1973)^113^
*Prevotella ruminicola*	Rumen, reported in swine cecum	3.62	47.7	Survival mechanism during periods of carbon starvation	Lou et al., (1997)^114^
*Ruminococcus albus*	Rumen	3.69	44.2	ND	Cheng et al., (1977)^115^
*Salmonella enterica*	Gut pathogen	4.86	52.2	Biofilm formation and virulence	Bonafonte et al., (2000), Levine et al., (1953)^91,116^
*Selenomonas ruminantium*	Rumen	3	50.7	Energy source in starvation conditions	Kamio et al., (1981), Wallace (1980)^117,118^
*Shigella dysenteriae*	Contaminated food and water, human gut pathogen	4.75	50.9	ND	Levine et al., (1953)^91^
*Streptococcus agalactie*	Human skin and mucous membranes (can be pathogenic)	2.28	35.8	Metabolic adaptation to stress conditions	McFarland et al., (1984)^119^
*Streptococcus mitis*	Human oral cavity and digestive tract; throat and nasopharynx	2.17	40	Survival	van Houte and Jansen (1970)^120^
*Vibrio cholerae*	Human gut pathogen	4.08	47.7	Environmental persistence and host transmission	Bourassa and Camilli (2009)^121^

a Genome size (Mb) of the reference genome of each specie.

b GC (%) of the reference genome of each specie.

### Biosynthesis and degradation of glycogen

Glycogen metabolism allows microorganisms to employ glucose polymers as a dedicated carbon and energy storage facility that can be mobilized for future purposes. This metabolic property relies on an enzymatic machinery responsible for glycogen biosynthesis and subsequent degradation, along with regulatory mechanisms in order to store or recruit glucose moieties depending on cellular needs.

In *E. coli*, *Bacillus megaterium* and other bacteria, glycogen is synthesized during times when carbon is abundant while other nutrients (e.g. nitrogen or phosphate) are limiting.^122,123^ However, in *Fibrobacter succinogenes* glycogen synthesis may take place during any growth phase.^111^ Glycogen accumulation in *L. acidophilus* is carbon source dependent.^112^ In contrast, glycogen degradation occurs when carbon sources become limiting.^43,124^ Under such circumstances, release of glucose units from glycogen by specific enzymes provide carbon resources to central metabolism.^125^ Therefore, analysis of the enzymatic functions involved in intracellular glycogen synthesis/degradation and their mechanisms of regulation are important to understand how and when microbes implement this apparent survival strategy.

**Figure 2.**
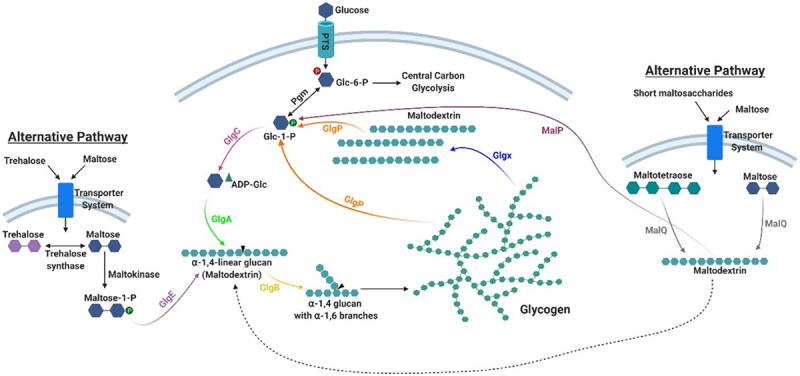
Glycogen metabolism pathways in gut commensal bacteria.

Most of the enzymes involved in glycogen metabolism in bacteria belong to glycosyl transferase or glycosyl hydrolase families, that contain specific carbohydrate-binding modules^87,88,126^ (Table 2). The classical glycogen biosynthetic pathway in bacteria, such as *E. coli* and *L. acidophilus*, involves phosphoglucomutase (Pgm), glucose-1-phosphate adenylyl transferase (GlgC), ADP-glucose-specific glycogen synthase (GlgA) and branching enzyme (GlgB). The enzymes GlgP (glycogen phosphorylase) and GlgX (debranching enzyme) are implicated in glycogen degradation (Table 2 and Figure 2).

Phosphoglucomutase (Pgm) converts glucose-6-phosphate into glucose-1-phosphate, which serves as a substrate for ADP-glucose synthesis catalyzed by GlgC in the presence of ATP and Mg^2+^. GlgA catalyzes the transfer of glucosyl units from ADP-glucose to the elongating chain of linear α-1,4-glucan with the concomitant release of ADP. Then, GlgB cleaves off portions of this α-1,4-glucan and links it to internal glucose molecules in existing chains via α-1,6 glycosidic bonds to form the glycogen structure. Glycogen catabolism is mediated by glycogen phosphorylase (GlgP) which removes glucose units from the non-reducing ends of the glycogen/maltodextrin molecules with concomitant phosphorylation, thereby liberating glucose 1-phosphate. At the same time, a debranching enzyme (GlgX) cleaves the α-1,6-bonds of the limit dextrins generated by GlgP releasing maltodextrins that can be further metabolized by GlgP. An alternative pathway employs trehalose or maltose to produce maltose 1-phosphate that is used by GlgE to elongate the glucan chain. Furthermore, enzymes involved in maltose/maltodextrin metabolism may play a role in glycogen synthesis and degradation. MalQ produces maltodextrins, using maltose or malto-oligosaccharides as substrates, and then GlgB can form glycogen from these linear maltodextrins. These maltodextrins can be cleaved by MalP liberating glucose-1-phosphate.

**Table 2.** Biosynthetic and degradative enzymes in the metabolism of glycogen.

Protein	Activity	EC number	GH/GT family*	Motif**
Pgm	Phosphoglucomutase	EC 5.4.2.2	^______^	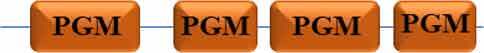
GlgC	Glucose-1-phosphate adenylyltransferase	EC 2.7.7.27	^______^	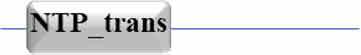
GlgA	Glycogen synthase	EC 2.4.1.11	GT5	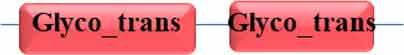
GlgB	1,4-alpha-glucan branching enzyme	EC 2.4.1.18	GH13, GH57	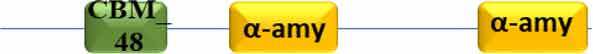
GlgP	Glycogen phoshorylase	EC 2.4.1.1	GT35	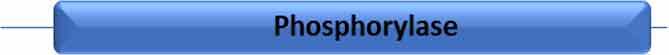
GlgX	Glycogen debranching enzyme	EC 3.2.1.68	GH13	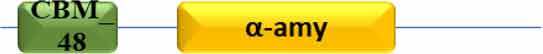
GlgE	Alpha-1,4-glucan:maltose-1-phosphate maltosyltransferase	EC 2.4.99.16	GH13	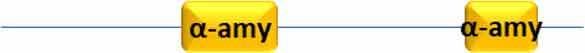
MalQ	4-alpha-glucanotransferase (amylomaltase)	EC 2.4.1.25	GH77	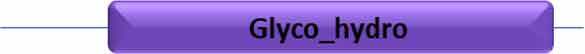
MalP	Maltodextrin phosphorylase	EC 2.4.1.1	GT35	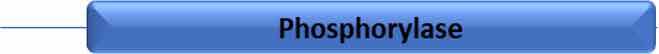

*GH Glycoside hydrolase; GT Glycoside transferase.

**PGM phosphoglucomutase; NTP_trans nucleotidyltransferase; Glyco_trans glucosyl transferase; CBM_48 carbohydrate binding module family 48; α-amylase; glyco_hydro glycoside hydrolase

Studies conducted on the gut bacterium *E. coli* were pivotal to elucidate the glycogen metabolic pathway that was described in detail by Preiss.^122,127^ Extracellular glucose is taken up and converted to glucose-6-phosphate by a bacterial phosphoenol-phosphotransferase system (PEP-PTS). Alternative sugars that can be converted to glucose-6-phosphate may also be used. Pgm converts glucose-6-phosphate into glucose-1-phosphate, which serves as a substrate for ADP-glucose synthesis catalyzed by GlgC.^127^ GlgA catalyzes the transfer of glucosyl units from ADP-glucose to the elongating chain of linear α-1,4-glucan with the concomitant release of ADP. GlgB subsequently cleaves off portions of this α-1,4-glucan and links it to internal glucose molecules in existing chains via α-1,6 glycosidic bonds to form the glycogen branching structure.^86^ Some bacteria possess a regulatory subunit of GlgC, called GlgD,^112^ where GlgC itself acts as the catalytic subunit. Glycogen catabolism is mediated by glycogen phosphorylase (GlgP) which removes glucose units from the non-reducing ends of the glycogen/maltodextrin molecules liberating glucose 1-phosphate.^128^ At the same time an isoamylase-type debranching enzyme (GlgX) cleaves the α-1,6-bonds of the limit dextrins generated by GlgP (typically 3–5 glucosyl residues in length) releasing maltodextrins^86,129^ that can be further metabolized by GlgP (Figure 2). The glucose-1-phosphate produced can be transformed into glucose-6-phosphate by Pgm and shuttled into the central carbon metabolic pathways, e.g. glycolysis or bifid shunt, the latter defined as the central and unique metabolic pathway for carbohydrate fermentation employed by bifidobacteria.^130^ This model represents the prevalent bacterial pathway of intracellular glycogen synthesis and degradation.^98^

It has been reported that certain microorganisms, such as species belonging to the genus *Streptomyces*, possess an alternative route for intracellular α-glucan biosynthesis, called the GlgE pathway. This alternative pathway employs trehalose or maltose as precursors for the disaccharide α-maltose 1-phosphate. A dedicated maltosyltransferase (GlgE) uses the latter molecule as the building block to elongate glucan chains^131,132^ (Figure 2). Furthermore, it has been described that maltose metabolic enzymes play a role in glycogen synthesis and degradation in *E. coli*.^133^ Briefly, the action of an amylomaltase (MalQ) on maltose or maltooligosaccharides can lead to the formation of maltodextrins^133^ and a maltodextrin phosphorylase (MalP) releases glucose 1-phosphate from glycogen molecules^90^ (Figure 2). Generally, the glycogen metabolism genes are organized in a single gene cluster in most bacteria known to accumulate glycogen (see section below).

### Presence and distribution of glycogen metabolism genes in human gut commensals

Genomics approaches have been crucial to reveal the complex interactions between a host and its resident bacteria and to unravel gut colonization strategies and microbiota functionality^46^. A previous survey of 55 bacterial genomes from different environments, such as plant, animals and humans, revealed that about half of these bacterial species contain an apparently full set of glycogen metabolic genes.^125,126^ This finding was later substantiated by another study of 1202 deduced bacterial proteomes.^134^ Based on recent genomic analyses, intact glycogen metabolic pathways are present in bacterial species adaptable to diverse environments and possessing flexible lifestyles.^98,134^ In contrast, a significant proportion of bacteria with parasitic behavior lack this glycogen biosynthetic ability.^126^ Taking advantage of the increasing number of available gut metagenomic datasets, we first examined the abundance of the glycogen metabolism pathway among selected members of the human gut community, using an existing computational method^135^ across 1,267 gut metagenomes from American, European, and Chinese subjects. We found evidence for the presence of this pathway in all metagenomes surveyed (minimum abundance = 1 copy/1000 cells), suggesting that this appears to be a core activity of the human gut microbiota (unpublished data).

We then analyzed the occurrence of putative glycogen gene products in 70 representatives of the different genera using the Catalog of Reference Genomes From the Human (gut) Microbiome^136^. We employed BLASTP analysis (cutoff values: E-value <0.00001, at least 20% identity across at least 50% of sequence length) and functional annotation profiling efforts to establish the occurrence of specific glycogen biosynthetic and degradative enzymes among gut bacteria (as outlined in Figure 2). Interestingly, this *in silico* analysis revealed that the majority of assessed members (~80%) of the gut microbiome, including components of the major bacterial phylogenetic divisions of the human gut microbiota, are predicted to harbor the metabolic genes involved in intracellular glycogen synthesis/breakdown, suggesting that this trait is a core function of such microbes from the gut environment. This analysis also revealed that the orthologous proteins are variably distributed across species, where certain enzymes seem to be more specific while others are present among all members. Therefore, there are organisms completely lacking *glg* homologs, organisms harboring partial sets of such homologs, and organisms harboring all known *glg* gene homologs, while some members of the latter two groups harbor more than one copy of a particular *glg* gene homolog.^98^

**Figure 3.**
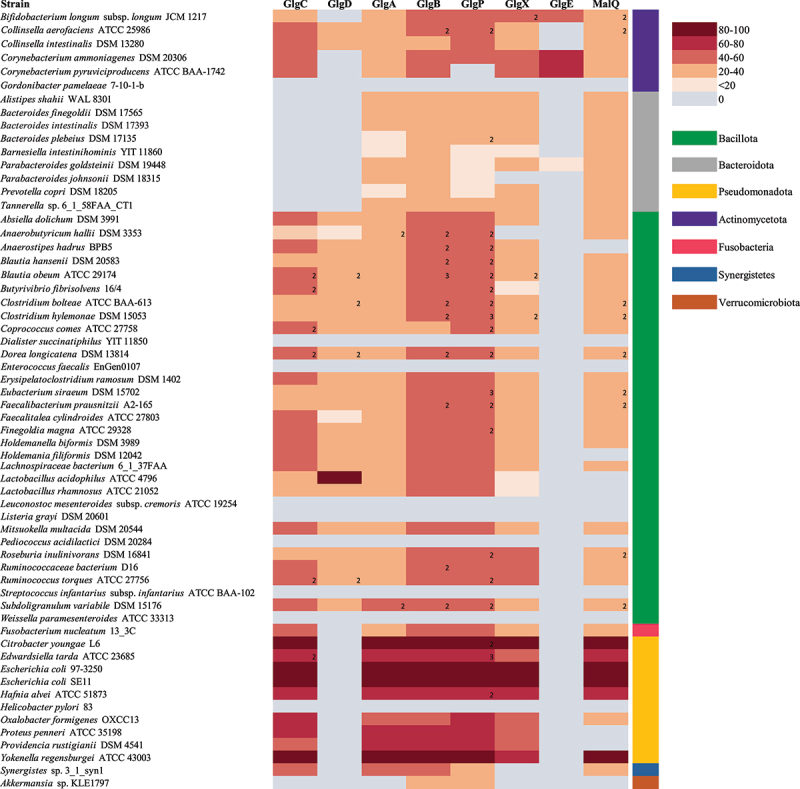
Presence/Absence of predicted glycogen biosynthetic and degradative enzymes in gut bacteria.

Remarkably, all members of Bacteriodetes lack GlgC though it may be that they use other proteins such as MalQ or UDP-glucose adenylyltransferase to generate the glycogen polysaccharide. Alternatively, Bacteroidetes members may not encode an *E. coli*-type GlgC enzyme, yet possess an enzyme that is more similar to human glycogen enzymes as has been described for other bacteria.^83^ Indeed, the glycogen synthesis pathway is functional in *Bacteroides fragilis* since accumulation of an intracellular polysaccharide formed from glucose has been demonstrated in 27 *Bacteroides fragilis* strains.^94^ Figure 3 shows that GlgE is not common in the adult human gut microbiome since only a relatively small number of species, for example members of the genus *Bifidobacterium*, encode this enzyme. Transcriptome analysis has revealed that the expression of genes predicted to encode proteins participating in glycogen metabolism are up-regulated under particular circumstances in *E. coli* and *L. acidophilus*.^88,112^ Nonetheless, bifidobacterial species that are not associated with the mammalian GIT lack genes encoding glycogen metabolism.^137^ Certain bacterial species that have been found in fecal samples, for example *Pediococcus acidilactici*, *Weissella paramesenteroides*, *Gordonibacter pamelaeae*, *Dialister succinatiphilus*, and *Leuconostoc mesenteroides* subsp. *cremoris*, among others, do not encode Glg proteins and thus do not appear to be able to synthesize intracellular glycogen. Some of these species may in fact have a food origin and therefore may not belong to the autochthonous gut microbiota population.^138^ The *glg* genes also appear to be absent from the genome of *Streptococcus infantarius* subsp. *infantarius* ATCC BAA-102, which is associated with colorectal cancer, or the pathogenic strains *Helicobacter pylori* 83 and *Enterococcus faecalis* EnGen0107 (Figure 3). As mentioned above, certain bacterial species contain more than one homologue of a particular *glg* gene, particularly in the case of *glgB* and *glgP*. Most notable examples are *Blautia obeum* ATCC 29,174 which possesses two homologues for *glgC*, *glgD*, *glgP* and *glgX*, and three *glgB* homologues, or *Dorea longicatena* DSM 13814 which contains two homologues for each of the *glgC*, *glgD*, *glgB* and *glgP* genes. In these species with several copies of a given *glg* gene, some of the copies represent homologs from different species (rather than being paralogs; data not shown). This peculiarity has been noticed previously in a study of glycogen metabolism genes in gammaproteobacterial species and may be a genetic adaptation to increase intracellular glycogen production/degradation rates or represent glycogen production under distinct circumstances by a specific set of *glg* genes.^98^

Heatmap representing the distribution across gut-related strains of proteins homologous to the enzymes involved in glycogen metabolism, using the protein sequences of GlgC, GlgA, GlgB, GlgP, GlgX and MalQ) from *E. coli* SE11, GlgD from *Lactobacillus acidophilus* ATCC 4796 and GlgE from *Corynebacterium glutamicum* DSM 20300 as query. Gene products from 70 representative strain genomes available on line from the Human (gut) Microbiome.^136^ The significant homology of 20% identity over 50% of protein length are represented in the matrix, employing a color code that represents the degree of identity. Members of the same phylum are indicated by the same color. The numbers indicate copies of a given protein. MalP and GlgP present a high identity of protein sequence and the BlastP analysis retrieved the same results using both sequences as query.

### Genetic organization of glycogen genes

**Figure 4.**
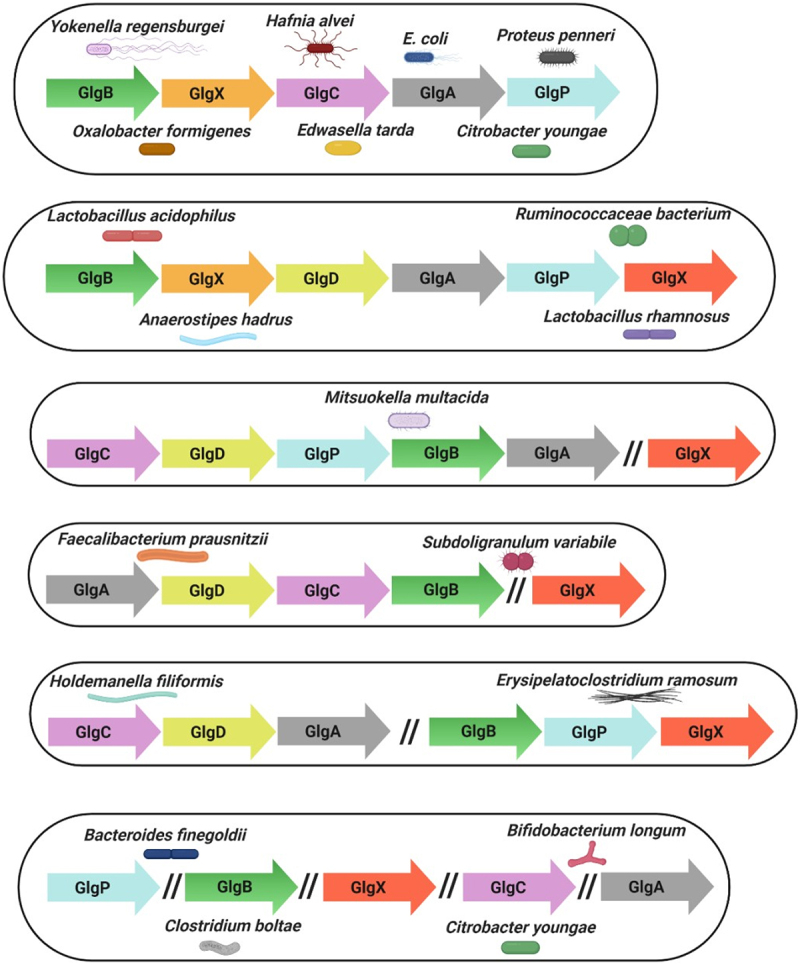
Genetic organization of glycogen genes in gut commensal bacteria.

Bacterial genes associated with the same metabolic pathway are often clustered.^98,139^ In *E. coli*, this cluster includes the *glgC*, *glgA* and *glgB* genes, plus two genes involved in glycogen degradation, *glgX* and *glgP*.^127,140^ The gene arrangement of these *glg* genes is *glgBXCAP* which is also conserved in all species harboring *glg* genes in the orders *Enterobacteriales* and *Pasteurellales*.^98^ Clustering of genes involved in glycogen metabolism has also been described for other bacteria, e.g. *Bacillus stearothermophilus* and *B. subtilis, Lactiplantibacillus plantarum* and *Vibrio* spp. among others.^86,98,103,141^ In the probiotic bacterium *L. acidophilus* the glycogen metabolic pathway is encoded by a 11.7-kb chromosomal region consisting of *glgBCDAP-amy-pgm*, while *glg* operons in other lactobacilli have the apparently conserved gene arrangement *glgBCDAP*^86,112^ (Figure 4). Notably, in the gut commensals surveyed in this review there are exceptions to this single *glg* gene cluster. For example in *Holdemanella filiformis* and *Erysipelatoclostridium ramosum* these genes are organized in two clusters, *glgCDA* and *glgBPX*. Curiously, in the species *Bacteroides intestinalis*, *Bacteroides finegoldii*, *Citrobacter youngae*, *Parabacteroides goldsteinii* and *Bifidobacterium longum* subsp. *longum*, among others, the *glg* genes are scattered across the chromosome (Figure 4).

Schematic representation of the gene arrangements of some bacteria, as indicated, containing glycogen genes identified in this review. The genes with identical function are represented by the same color.

Thus, the presence of complete glycogen pathways, and their tight regulation, in human gut commensals is likely reflecting the importance of this metabolism in the gut ecosystem (see Box 2 and next section).

### Box 2: Regulation of glycogen metabolism

Glycogen metabolism is closely interconnected with various metabolic processes as reviewed by Wilson and colleagues.^88^ Thus, not surprisingly, it is subject to complex transcriptional, post-transcriptional and allosteric regulation as described below. The reader should be aware that most of the findings related to regulatory mechanisms of glycogen metabolism were generated in *E. coli* and will be covered as such in the following paragraphs unless otherwise indicated.

At a transcriptional level, *glg* genes are generally co-ordinately expressed within various regulons, and their expression is triggered by signals related to nutrient starvation. Carbon catabolite repression (CCR) is a transcriptional regulatory mechanism aimed at ensuring sequential utilization of more preferred carbon sources through repression of genes required for the metabolism of less preferred carbon compounds.^142^ This repression has been associated with activation of glycogen metabolism upon glucose shortage in several organisms^142^ including *E. coli, Salmonella spp., Streptococcus suis*, *Corynebacterium glutamicum*, *Mycobacterium* spp., or *V. cholerae*.^47^ So-called catabolite responsive elements (*cre*) are located upstream *glg* genes in various organisms, which indicates CCR-mediated regulation.^143^ Glucose and other nutrient depletion signals also trigger the stringent response, which re-programmes metabolic processes allowing bacteria to adapt and survive adverse conditions.^144^ In *E. coli* the stringent and general stress responses are major determinants of intracellular glycogen content.^145^ Under a range of stress factors and nutrient limitations, the alarmone ppGpp, a small nucleotide that acts as a global transcriptional regulator in bacteria, is a well-established positive regulator of the glycogen biosynthetic genes in *E. coli*.^146–148^ However, glycogen accumulation is independent of (p)ppGpp or *cre* elements in various organisms, suggesting that these genes are subject to an alternative control mechanism.^145^ Alternative sigma factors have been implicated in the transcriptional control of *glg* genes in *E. coli, Bacillus subtilis* and *C. difficile*.^103,149,150^

At post-transcriptional level, glycogen storage is negatively regulated by the carbon storage regulator CsrA, a small-RNA binding protein that inhibits expression of targeted genes^151,152^. CsrA controls a wide variety of cellular processes including some that affect interactions of enteric species with mammalian hosts, such as central carbon metabolism, biofilm formation, virulence and motility, among others^42,153,154^. At the enzymatic level, allosteric mechanisms of regulation have been described for GlgC and GlgP, which catalyze key steps in the biosynthesis and catabolism of intracellular glycogen, respectively. Specifically, these enzymes are generally activated by metabolites that reflect signals of high carbon and energy content such as phosphoenolpyruvate, glucose-6-phosphate, fructose-1,6-bisphosphate or pyruvate, whereas they are inhibited by metabolites reminiscent of low metabolic energy levels like AMP, ADP and/or Pi^141,145,155–159^. While different levels of allosteric regulation for these enzymes have been described in various organisms^141,157^, allosteric regulation of GlgC is considered a main control point of glycogen accumulation in early stationary phase cells^160^.

## Roles of glycogen storage in gut bacteria

Environmental stresses in the GIT impose physiological challenges upon bacteria^161^ which must adapt in response to such adverse conditions^29^ (see Box 1). In this regard, glycogen accumulation is a common feature across phylogenetically divergent species, and appears to represent a bacterial adaptation to nutrient (or other) limitation in the gut.

What follows below is an in-depth analysis of available evidence, obtained from *in vitro* studies simulating the gut environment and *in vivo* studies employing animal models. All of which point toward glycogen metabolism as a widespread and fitness-enhancing trait among gut commensals.

### The role of glycogen accumulation in simulated gut conditions

Glycogen is a stored energy resource linked to important physiological functions in bacteria as discussed below. Transient and resident gut commensals, including those administered as adjunct cultures in fermented foods or those provided as probiotic supplements, must cope with a range of challenges during their transit along the GIT that serially enforce strong selective pressures hampering bacterial survival and establishment in the lower gut ecosystem.^162,163^ Glycogen accumulation has been linked to (long-term) survival where it can offer an energy source when the gut environment cannot.^88^ However, the role of glycogen metabolism as a specific strategy for gut commensals to survive and thrive in their natural habitat has not received much scientific attention. Next, laboratory *in vitro* experiments simulating various stresses are shown to provide valuable insights to understand processes that occur in the mammalian gut^164^.

#### Glycogen storage and survival upon macro- and micro-nutrient starvation

Nutrient limitation will for obvious reasons affect the gut microbiota. Nutrient availability in the GIT is affected by circadian oscillations, imposed by periods of fasting and feeding,^29,43,165,166^ and is in general characterized by the presence/absence of non-digestible complex carbohydrates as carbon sources.^167^ Under such circumstances, metabolic flexibility and mixed-substrate utilization offer selective strategies for efficient gut colonization and survival.^29,168^ These circumstances include: (i) availability of complex and non-digestible carbon sources, (ii) ability or lack thereof to utilize metabolites or sub-products released by other neighboring gut microbial inhabitants, and (iii) an ability to efficiently adapt metabolic activity to available nutrients where and when appropriate.

Several *in vitro* studies have investigated if the ability to accumulate glycogen affects survival of gut commensals under carbon starvation. For example, it was demonstrated that *En. aerogenes* cells containing glycogen survived better in an unfavorable environment in which carbon was limited.^169^ In *L. acidophilus*, cells showed stable intracellular glycogen reserves during prolonged growth periods and for this reason it has been speculated that *L. acidophilus* maintains a higher level of intracellular glycogen to enhance its residence time when growing under conditions simulating the GIT environment.^112^ Mutations that disrupt glycogen metabolism were also shown to negatively impact on *E. coli* fitness during nutrient starvation, demonstrating its role in maintenance of viability and alternative energy source acquisition during metabolic transitions.^42^

These publications suggest that under carbon deprivation, glycogen is utilized to preserve cell viability, providing the energy and carbon source required by the bacteria for maintenance.^48^ This bacterial durability has been linked with glycogen average chain length: glycogen with an average short chain length correlated with prolonged bacterial survival.^134^ This effect was attributed to the slower degradation of this short chain length polymer, which can sustain bacterial viability for prolonged periods of time.^125^

Regarding nutrient competition, the presence of specific alternative carbon sources to glucose, such as raffinose and the disaccharides trehalose and lactose, has been described to act as a signal promoting intracellular glycogen accumulation in *L. acidophilus*.^86^ Raffinose is a non-digestible carbon source, known to be specifically fermented at intestinal level by several commensal species including representative strains of lactobacilli and bifidobacteria.^170^ Specifically, growth on raffinose was associated with temporally increased expression of *glg* genes, which was shown to coincide with glycogen accumulation. Furthermore, glycogen accumulation was required to support *L. acidophilus* growth on raffinose as *ΔglgA* and *ΔglgB* mutants were shown to exhibit impaired growth on this carbohydrate.^86^ These results suggest a close relationship between the metabolism of certain carbohydrates and glycogen metabolism, and although the specific molecular connections are currently not understood, both pathways are likely to be under the control of common central carbon regulators (see section about regulation in Box 2). Indeed, evidence in lactobacilli suggests that *glg* expression and glycogen accumulation are repressed by glucose as in other enteric bacteria, thus its expression might be enhanced in the presence of other carbon sources such as the ones typically encountered within the gut ecosystem. Thus, glycogen synthesis seems to be triggered by certain environmental conditions despite high energy and carbon availability, while glycogen degradation occurs during extended nutrient starvation.^171^

The limitation of micronutrients in the gut may also affect intracellular glycogen metabolism in bacteria. For example, the bacterial response to a shortage of phosphate in the gut, which can occur under physiological stress conditions of the host, includes activation of virulence in a broad range of pathogens, as well as of glycogen accumulation.^172^ Nitrogen is another limiting nutrient for gut microbial communities, that strongly determines gut microbial composition and their interactions with the host.^173,174^ For example, *E. coli* MG1655 cells grown under nitrogen-limiting conditions were shown to exhibit the highest level of glycogen accumulation compared with carbohydrate-limited conditions during stationary phase.^108^

Remarkably, a shortage of nitrogen has been demonstrated to activate the stringent response, evidenced by increased glycogen accumulation in *E. coli* and *Vibrio cholerae* cells grown in the presence of glucose excess.^121,175^ In *E. coli*, limited availability of iron also connects the stringent response to glycogen metabolism as it promotes ppGpp accumulation which overall results in promotion of glycogen biosynthesis.^176^ Accordingly, mutants defective in sensing and sequestering iron display altered glycogen accumulation phenotypes.^145^ Consistent with this observation, it was found that the *glgX* gene of the commensal *Bifidobacterium breve* UCC2003 is important for survival under iron-limiting conditions.^177^

Thus, glycogen accumulation as a means of intracellular carbon and energy storage may represent a common survival mechanism for commensals across different compartments of the GIT and an advantage in an environment with strong competition for available nutrients.

#### Glycogen storage and Survival upon acid and bile stress conditions

It is increasingly clear that many stress responses overlap and thus a stress response to one specific factor commonly results in cross-resistance to other, apparently unrelated stressors.^178^ In this regard, it is conceivable that glycogen accumulation, when activated in response to decreased availability of nutrients and/or micronutrients, plays a role in the response and tolerance to other stress factors encountered in the GIT. As an example of this interconnection, nutrient starvation, which generally marks entry into the stationary growth phase, also represents the activation signal for glycogen accumulation.^179^ Besides, it is well known that cells in the stationary phase are significantly more tolerant to acid stress than those in the log phase of growth.^180^ It is also worth noting that some of the regulators controlling glycogen metabolism, control the expression of genes that contribute to the response to specific GIT stress factors, suggesting that intracellular glycogen metabolism may confer a selective advantage in order to better cope with specific GIT stress factors. The specific contribution of glycogen metabolism toward acid tolerance has not been investigated, although glycogen reserves act as intracellular energy sources, which are required for the maintenance of pH homeostasis under acidic conditions supported by the extrusion of protons via the F_0_F_1_ H^+^/ATPase.^181^ Thus, under acidic conditions, cells tend to adjust their metabolism, by increasing the synthesis of energy-rich intermediates (such as ATP and NADH) and/or glycogen.^182^ A connection between acid and bile stress responses has also been proposed as intracellular accumulation of BAs leads to proton release and thus cytoplasmic acidification. Early reports have described that bile salts promote higher intracellular accumulation of glycogen in several enterobacteria.^91^ For example, response to and survival under acidic conditions in *E. coli* is highly dependent on the alternative σ^S^ factor, RpoS, also known to control glycogen accumulation.^150^ Furthermore, the AcrAB-TolC multidrug efflux system from enterobacteria, essential for bile resistance in *Salmonella typhimurium*, is activated as part of the RamA-controlled regulon, which is also known to regulate *glg* genes in other microorganisms.^183,184^

More recently, *glg* gene inactivation in the commensal *L. acidophilus* did not appear to affect survival in simulated gastric juices, indicating the absence of a role in acid tolerance, although it was shown to negatively affect survival in simulated intestinal juices, suggesting a role in survival to bile. Indeed strains carrying a *ΔglgB* or *ΔglgP* mutation were shown to elicit growth defects, including longer lag phases during growth in the presence of bile extracts, further supporting a contribution of this metabolic pathway in the gut ecosystem.^86^

#### The role of glycogen accumulation in biofilm formation

In nature bacteria are rarely encountered in planktonic lifestyles but are organized in matrix-enclosed communities known as biofilms. Recent findings suggest that bacteria within the gut environment are mainly encountered in the form of intricate biofilms that establish close association with the intestinal epithelia.^185^ Besides, biofilm lifestyles confer resistance to harsh environments thereby rendering biofilm-associated bacteria more resilient to environmental challenges such as those encountered in the gut. In enteric bacteria, *in vitro* studies have demonstrated a correlation between glycogen accumulation and biofilm formation. Similarly, studies in *E. coli* have revealed that biofilm formation is enhanced in mutants defective for CsrA, a global carbon regulator that also represses glycogen storage during active phases of bacterial growth. Besides, it has been reported that biofilm formation phenotypes are affected in mutants with altered glycogen accumulation and subsequent catabolism, as illustrated by the finding that in *E. coli* glycogen accumulation and catabolism through GlgP is required to promote biofilm formation.^186^ Consistent with this notion, in some bifidobacteria, in which biofilm production has been demonstrated to occur in response to bile exposure, a *glgP* insertional mutant (a mutation expected to prevent glycogen degradation) rendered the strain defective in biofilm formation when exposed to high bile concentrations.^187,188^ However, it is worth noting that contradictory observations have been reported as a glycogen-deficient *ΔglgC* (glycogen synthesis) mutant was shown to accumulate high levels of biofilm in *E. coli*.^189^ These observations clearly indicate that, glycogen metabolism and, particularly, degradation, affect biofilm formation. Further investigation is required so as to decipher the regulatory mechanisms connecting biofilm formation and glycogen metabolism although it has been speculated that glycogen catabolism can serve as energy and/or carbon source to promote biofilm formation.^162^ In agreement with this hypothesis, the ability to utilize glycogen was demonstrated to be important for the transition between planktonic and biofilm lifestyles and enabled increased glucose uptake during pulses of limited glucose availability in *E. coli*.^161^

## Role of glycogen storage for colonization and fitness in the gut ecosystem

Deciphering the functions of gut microbiota members is important to understand the factors driving colonization, resilience or survival in the gut niche.^29^ Since glycogen accumulation plays a role in survival under *in vitro* conditions, this bacterial metabolism is also thought to be a crucial mechanism involved in GIT colonization and fitness. Thus, this section focuses on several *in vivo* studies performed to determine the impact of bacterial glycogen storage on enhancing fitness in the gut ecosystem. It is possible that glycogen storage plays an important role in GIT colonization due to the necessity to support growth and/or to maintain viable in the intestinal environment when and where there is intense competition for (micro)nutrients.

The ability of *L. acidophilus* to synthesize and store glycogen was shown to support long-term colonization in the murine GIT.^86^ In this *in vivo* study two bacterial strains, the parent *L. acidophilus* (able to synthesize and store glycogen) and its *glgA* mutant unable to synthesize glycogen were administered in a 1:1 ratio to germ-free (GF) mice. The results showed that colonization levels of the *glgA* mutant are significant lower compared to the parental strain for almost one month post-delivery. These findings represent compelling evidence that the ability of *L. acidophilus* to synthesize intracellular glycogen provides a competitive advantage that supports gut colonization. In the same study, the authors also showed that a *glgA* mutant is able to colonize the gut of GF mice in the absence of competition. However, when the parental strain was administered to the mice colonized with the *glgA* mutant the decline in the mutant abundance coincided with an increase of the parental population level. This supports the idea that intracellular glycogen storage plays a major role in the competitive fitness of *L. acidophilus* in the host environment. The importance of glycogen accumulation in murine GIT colonization has also been demonstrated using the commensal *E. coli* K-12. It has been shown that *E. coli* mutants which lack genes for glycogen accumulation are not able to colonize the murine GIT. However, by providing a constant supply of a readily metabolizable sugar in the animal’s drinking water, the competitive disadvantage of *E. coli* glycogen metabolism mutants was rescued, supporting the notion that glycogen storage may aid to maintain fitness when there is intense competition for resources and occasional nutrient deprivation.^43^

The trait of glycogen storage in an ecological context has also been addressed by metagenomic and metatranscriptomic approaches. Metagenomic analysis can provide host-specific insights into diversity and functional potential of the gut microbiota. Studies such as the mouse intestinal bacterial collection (miBC) identified factors required for colonization in mice. This study described the presence of glycogen genes in the strains *Blautia caecimuris*, *Cuneatibacter caecimuris*, *Extibacter muris*, *Irregularibacter muris*, *Longicatena caecimuris* and *Pasteurella caecimuris* among others.^190^ A metatranscriptomic study in *Bacteroides* defined *in vivo* fitness determinants for these gut members in a community context under different dietary conditions. Genes involved in glycogen storage were significantly upregulated in *Ba. ovatus*, *Ba. cellulosyliticus* and *Ba. thetaiotaomicron*, during intestinal colonization in mice. Thus, these genes appear to be important for colonization and survival in a given environmental context^191^. In another study involving the ruminal microbiome, the glycogen phosphorylase-encoding gene, among other functional features, was highly transcribed by 115 bacterial genera, mainly belonging to the phyla Pseudomonadota, Bacillota, and Actinomycetota.^192^

Much information can be gained from the distribution of genes that are functionally important in complex microbial communities by targeting specific genes.^193,194^ Using targeted metagenomic sequencing, the gene encoding the GlgB enzyme (which is crucial for glycogen storage and metabolism)^195^ was found to be present in 69 gut microbiomes from human, pig, cow and chicken hosts.^196^ For example, *glgB* sequences in human gut microbiota members included *Clostridia* sp., *Faecalibacterium* sp. and Lachnospiraceae. The activity of *glgB* in the gut, and their influence on microbial dynamics, are likely to be associated with diet, as carbohydrates are indeed the direct target of this enzymatic activity.^196^ Remarkably, a metatranscriptomic analysis of the human gut microbiome revealed that specific genes involved in carbohydrate metabolism, including *glgB* (glucan branching enzyme), were highly expressed by Fusobacteria. These data indicate that important functional aspects of low abundance bacterial species are active contributors to the gut ecology and host physiology.^197^ In this regard, the pathway for glycogen synthesis was shown to be active in the gut by members of *Escherichia*, *Sutterela*, *Enterobacter* and *Enterococcus*.^198^

Another metatranscriptomic study on metabolic adaptation of the human gut microbiota during pregnancy and infancy revealed that *glgC*, involved in glycogen synthesis, was upregulated in the maternal microbiota prior to birth.^199^ It has also been demonstrated that in many bacterial species synthesis of glycogen is stimulated when glucose is abundant in the medium^123^. High glucose concentration in the gut environment can be found during the state of late pregnancy, where the ability of microbiota to transport and degrade a variety of carbohydrates is replaced by the overexpression of a single transporter capable of internalizing glucose, and of enzymes of the glycogen synthesis pathway.^199^

All the above indicates that there is a correlation between the presence of *glg* genes and their expression among members of the gut ecosystem, supporting the notion that glycogen metabolism is an important fitness property for gut bacteria.

## Glycogen and gut pathogenic bacteria

It has been suggested that glycogen storage increases bacterial virulence and survival at mucosal surfaces, although this view is still controversial. For example, it has been demonstrated that the ability of glycogen accumulation in *Salmonella gallinarum* and *Salmonella pullorum* does not confer a fitness increase but appeared to enhance long-term survival.^200^ On the other hand, it has been shown that glycogen accumulation in *Salmonella enterica* is important for biofilm formation to increase its virulence capacity.^116^ The pathogen *V. cholerae* is able to store glycogen *in vivo* and this mechanism plays an important role in the transition of *V. cholerae* between poor nutrient environment (such as an aquatic niche) and the host, because mutants that are either unable to store or degrade glycogen are attenuated for transmission.^121^ Genome analysis of *Clostridioides difficile* comparing *in vitro* versus *in vivo* conditions revealed that the genes *glgA*, *glgC* and *glgD* (*glgCDA* operon) are upregulated under *in vivo* conditions, suggesting that *C. difficile* accumulates glycogen under such circumstances.^201^ The latter work also indicated that glycogen accumulation in *C. difficile* is one of the strategies used by this bacterium to cope with temporary starvation conditions typical of gut colonization.^201^ To further explore the possible link between glycogen storage and *C. difficile* virulence a mutant of this bacterium lacking *glgC* was shown to produce significantly fewer spores compared to the parental strain, indicating that glycogen storage is required for efficient sporulation in *C. difficile*.^202^ While more studies are needed to address the precise role of bacterial glycogen storage *in vivo* under various conditions of host environmental nutrient and metabolic perturbation are needed, currently available data indicate that this mechanism is used as a strategy to survive challenges encountered in the host environment. Sporulation is a mode of long-term survival that has been described as a widespread strategy used by gut bacteria to survive under nutritional limitations or other stressful conditions, and to transit from the environment to the host.^203^ Thus, in these bacteria which experience frequent oscillations of their environmental conditions, in particular when they transfer from a non-host environment to the gut of their host, glycogen accumulation is essential to survive outside the host.^121,204^ In conclusion, commensal or pathogenic bacteria that are able to synthesize and store glycogen survive better and longer than their isogenic derivatives that don’t possess the ability to build up such energy and carbon reserves.

## Concluding remarks

**Figure 5.**
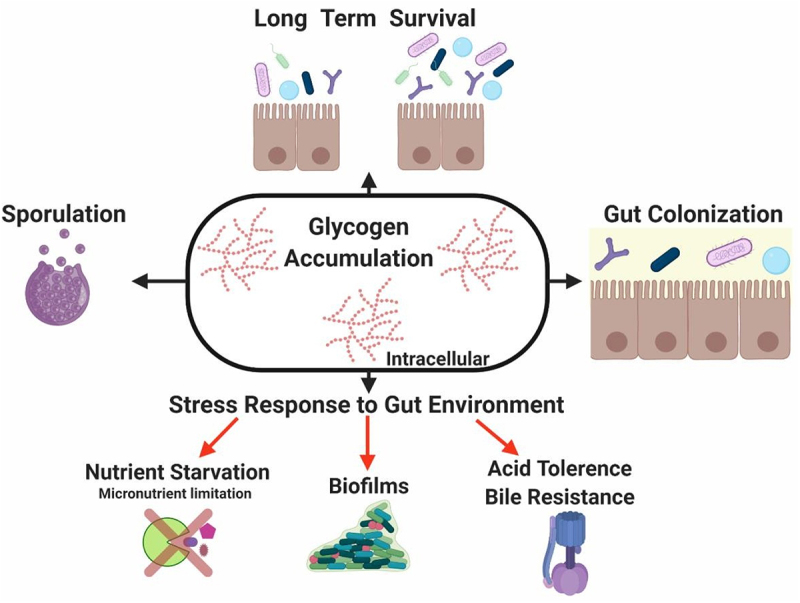
Role of glycogen metabolism in gut commensal bacteria.

Determining the functional attributes of the microbiome is essential to understand its role in host physiology, metabolism and disease and it has also prompted a wide range of biotechnological applications to improve human health. This includes the production of novel probiotics and prebiotics, among others, to modulate the gut microbiota. However, these technological developments require an in-depth knowledge of the adaptive mechanisms of these microbial communities to the human GIT. In this regard, these microorganisms must have developed metabolic strategies to efficiently colonize and survive in such a harsh, dynamic and highly competitive environment. The genetic capability for glycogen metabolism as present in a large proportion of enteric bacteria, supports the notion that this metabolic pathway enhances adaptation and performance of gut commensals in their specific ecosystem. Not surprisingly, accumulation of this polymer is interconnected to central carbon metabolism and several fundamental biological processes as demonstrated by its intricate regulatory mechanisms. Available information suggests that the ability to synthesize and store intracellular glycogen by gut bacteria is linked to their long-term survival, adaptation to periods of starvation, protection against a range of stressors relevant to the gut ecosystem while also being important, and in some cases essential, for colonization of the GIT (Figure 5).

Glycogen accumulation confers several potential advantages to bacteria enabling them to persist and survive into the gut environment. Among others, this metabolic process can: i) mediate sporulation, a strategy used by gut bacteria to survive under life-threatening conditions; ii) ensure long-term survival in periods of famine; iii) facilitate gut colonization; iv) efficiently response to stress conditions such as nutrient starvation, biofilm formation and promote acid tolerance and bile resistance.

This metabolic feature shapes functionality and composition of gut microbiome from a novel perspective. While most of the findings in this field are based on *in vitro* studies, the role of glycogen metabolism from an ecological perspective has been addressed in this review including metagenomics and metatranscriptomics of complex microbial communities and *in vivo* studies. Functional understanding and delineating the diversity, extent and regulation of this metabolic pathway in the gut microbiota may provide novel clues to improve their performance within the gut ecosystem.
